# The impact of Benevolent Childhood Experiences on adult Flourishing: the mediating role of Light Triad traits

**DOI:** 10.3389/fpsyg.2024.1320169

**Published:** 2024-04-24

**Authors:** Miguel Landa-Blanco, Tatiana Herrera, Helen Espinoza, Kilver Girón, Samantha Moncada, Antonio Cortés-Ramos

**Affiliations:** ^1^School of Psychological Sciences, National Autonomous University of Honduras, Tegucigalpa, Honduras; ^2^Faculty of Education and Sport Sciences, University of Granada, Melilla, Spain

**Keywords:** Benevolent Childhood Experiences, Flourishing, Light Triad, Humanism, Faith in Humanity, Kantianism

## Abstract

The literature has well documented the relationship between Adverse Childhood Experiences, personality traits, and well-being. However, less is known about how Benevolent Childhood Experiences (BCEs) relate to “light” personality traits and Flourishing. The study analyzed the effects of BCEs on Flourishing, considering the mediator role of Light Triad traits (Kantianism, Humanism, and Faith in Humanity). The study used a quantitative methodology with a non-experimental, cross-sectional design; 410 Honduran adults responded to the survey, including questions regarding Light Triad personality traits, Flourishing, and BCEs. On average, respondents reported 7.34 BCEs. The number of reported BCEs did not vary significantly between men and women. However, specific BCEs were categorically associated with subjects’ sex. A higher proportion of men reported having at least one teacher who cared about the respondent, having opportunities to have a good time, and liking/feeling comfortable with oneself. Flourishing was significantly higher for participants who reported the presence of BCEs. The largest effect size was achieved for the difference in Flourishing scores between those who reported liking school as a child and those who disliked it. The number of Benevolent Childhood Experiences had a significant total and direct effect on Flourishing scores. Significant indirect effects were also identified. Faith in Humanity and Humanism, not Kantianism, mediated the relationship between BCEs and Flourishing. BCEs significantly explained all Light Triad traits. In conclusion, BCEs have significant direct and indirect effects on adult Flourishing; Faith in Humanity and Humanism mediate this relationship.

## 1 Introduction

Mental health encompasses well-being, enabling individuals to confront vital challenges and fulfill their potential. The concept extends beyond the absence of psychopathology (WHO, 2022), as it also englobes human strengths, positive emotions, and subjective well-being (Vaillant, 2012); this is often referred to as positive mental health. Psychotherapists consider positive mental health screening and interventions innovative and valuable assets for the therapeutic process (Chang et al., 2022).

Childhood experiences have diverse outcomes in various adulthood domains, including physical and mental health and family well-being (Crandall et al., 2019; Daines et al., 2021; Mosley-Johnson et al., 2021). These childhood experiences can be either benevolent (positive) or adverse (negative). Research has shown how Adverse Childhood Experiences (ACEs) are associated with adult personality traits. Specifically, ACEs are negatively related to conscientiousness and positively associated with neuroticism (Grusnick et al., 2020). Such experiences are also positively related to “dark” traits, including psychopathy, borderline personality disorder, and narcissism (Wilson et al., 2023), as well as lower subjective well-being (Wang et al., 2022). Therefore, there is clear evidence of the relationship between Adverse Childhood Experiences, “dark” personality traits, and well-being. However, less is known about how Benevolent Childhood Experiences (BCEs) relate to “light” personality traits and Flourishing.

Benevolent Childhood Experiences include the social and family support a person receives before turning 18; they also involve comfortable beliefs, opportunities for having a good time, stable home routines, self-acceptance, and school enjoyment (Narayan et al., 2018). Recent studies suggest that BCEs predict lower symptoms of depression, stress, and loneliness during adulthood. It is worth noting that BCEs are significant mental health promoters independent of ACEs (Doom et al., 2021).

The Light Triad (LT) of personality focuses on positive traits: Humanism, Faith in Humanity, and Kantianism. Humanism refers to the belief that people are inherently worthy and have dignity. Faith in Humanity is a predisposition to focus on the best in people and to believe that most people are good. Kantianism is the belief that people are ends unto themselves. Previous studies suggest that such traits are positively related to life satisfaction (Kaufman et al., 2019).

On the other hand, Flourishing refers to a multidimensional state of optimal psychological well-being characterized by positive emotions and relationships, engagement in activities, a sense of meaning and purpose, and a sense of accomplishment and personal growth. It represents a holistic and positive perspective on well-being, focusing on cultivating and enhancing individuals’ positive functioning and overall quality of life. Flourishing extends beyond the absence of negative symptoms or disorders, emphasizing promoting positive attributes and experiences (Diener et al., 2010). As such, it is considered an essential aspect of mental health promotion (Burns et al., 2022). Previous studies have shown that experiencing parental warmth during childhood is significantly associated with Flourishing scores during mid-adulthood (Chen et al., 2019); suggesting a link between childhood experiences and adulthood subjective well-being (Yu et al., 2022).

Studying BCEs in a specific cultural context, such as Honduras, enhances our understanding of how cultural factors shape childhood experiences and their impact on adulthood. Cultural values, norms, and practices play a crucial role in shaping the experiences and perceptions of individuals. By examining BCEs within the Honduran context, researchers can identify culturally specific factors contributing to positive childhood experiences and their potential long-term effects on individuals’ well-being. Research conducted in diverse cultural contexts helps us move beyond a narrow focus on Western perspectives and provides a more comprehensive understanding of the universal and culturally specific factors contributing to positive childhood development.

In this sense, Honduras has been considered one of the most violent countries in the world. Young people are at high risk of being victims of violence (Landa-Blanco et al., 2020). Poverty and illegal migration are also prevalent in the country. Many children suffer traumatic experiences before, during, and after the migratory process; it is common for children to migrate unaccompanied (Linton et al., 2018). Most young adults sampled in a national study have experienced ACEs (Huber-Krum et al., 2022). Women were at a higher risk of reporting ACEs related to sexual, emotional, or physical violence. Experiencing ACEs was related to a greater prevalence of depression, distress, and suicide risk (Kappel et al., 2021). Recent studies from Honduras have also reported that certain ACEs exhibit notably detrimental effects on mental health outcomes. These include instances of coerced sexual activity, exposure to domestic violence within the family, verbal degradation, and residing with individuals grappling with mental health challenges, substance abuse problems, or incarceration histories (Landa-Blanco et al., 2024).

While these studies offer valuable insights, they tend to adopt a psychopathology-focused approach, overlooking key dimensions of the well-being spectrum. Therefore, from a positive mental health approach, the current study analyzed how Benevolent Childhood Experiences affect Flourishing, considering the mediator role of Light Triad personality traits in the Honduran population. Taking into account the literature review presented here, the following hypotheses were established:

- *Hypothesis 1*: Benevolent Childhood Experiences have positive direct effects on Light Triad traits.
- *Hypothesis 2*: Benevolent Childhood Experiences have positive direct effects on Flourishing.
- *Hypothesis 3*: Benevolent Childhood Experiences have positive indirect effects on Flourishing, Light Triad traits mediate this relationship.

## 2 Methods

### 2.1 Participants

A total of 410 participants responded to the survey; 255 (62%) were women, and 155 (38%) were men. Their average age was 27.26 years (*SD* = 10.49, Min = 18, Max = 68). Subjects were non-probabilistically selected through convenience and snowball sampling. The online survey was disseminated through emails, social media, university classrooms, etc. The inclusion criteria were: 1) being 18 years or older, 2) being from Honduras, 3) currently living in Honduras, and 4) agreeing to the informed consent.

### 2.2 Instruments

#### 2.2.1 Benevolent Childhood Experiences

The Benevolent Childhood Experiences Scale consists of 10 categorical (yes = 1/no = 0) items (Narayan et al., 2018). Each question asks about specific positive occurrences experienced during the first 18 years of life, for example: “Did you have at least one caregiver with whom you felt safe?,” “Did you like school?,” “Did you have a predictable home routine, like regular meals and a regular bedtime?,” among others. Summative BCE scores were calculated; higher totals indicate a higher number of BCEs (Min = 0, Max = 10). The scale has adequate reliability (*ω* = 0.70).

#### 2.2.2 Light Triad of personality

The Light Triad Scale (LTS) measures three distinct personality traits through 12 items (Kaufman et al., 2019): 1) Humanism, 2) Faith in Humanity, and 3) Kantianism. Sample items include: “I enjoy listening to people from all walks of life,” “I tend to applaud the successes of other people,” among other affirmations. Each one is rated on a 5-point Likert Scale (1 = totally disagree, 5 = totally agree), with higher summative scores indicating a higher intensity of the trait. Based on the current data, the LTS had a good internal consistency (*ω* = 0.85).

#### 2.2.3 Flourishing

The Flourishing Scale (FS) is an 8-item unidimensional questionnaire (Diener et al., 2010). Responses are presented in a 7-point Likert-type questionnaire (1 = totally disagree; 7 = totally agree). Higher summative scores (*Min* = 8; *Max* = 56) indicate a higher self-reported Flourishing. Some items included in the FS are: “I am competent and capable in the activities that are important to me,” “I lead a purposeful and meaningful life,” among others. The FS achieved adequate psychometric properties in the Honduran population, including a high internal consistency (McDonald’s *ω* = 0.89), test–retest reliability, convergent and divergent validity, as well as an unidimensional factor structure (Landa-Blanco et al., 2023).

### 2.3 Ethical considerations

This study adhered to ethical guidelines to ensure the protection and welfare of the participants. Before participation, subjects were provided with a clear explanation of the study’s purpose, procedures, risks, benefits, and their right to withdraw during the data collection. They were informed that their participation was voluntary. The questionnaire used in this study was designed to maintain the anonymity of the participants; no personally identifiable information was collected during the data collection process. Results were reported at the group level, and no personally identifiable information was disclosed in any form of dissemination.

At the beginning and end of the survey, participants were presented with a link to the “UNAH Te Escucha,” an online real-time chat platform that provides psychological assistance to the Honduran population. The School of Psychological Sciences of the National Autonomous University of Honduras (UNAH) runs this free-of-charge website. The study was approved by the Research Ethics Committee of the Faculty of Social Sciences of the UNAH (Macrostudy-CEIFCS-P1-2023).

### 2.4 Data analyses

First, summative scores were calculated for all variables. The internal consistency of each scale was determined using McDonald’s *ω*. Then, descriptive statistics were used, specifically, Mean (*M*) scores, Standard Deviations (*SD*), absolute and relative frequencies. Two-group comparisons were made using Welch’s *t*-test and Cohen’s *d* (as effect size estimate). Categorical associations were determined by a chi-square test (*χ^2^*) and Contingency Coefficients (*CC*). Pearson’s *r* coefficient was used to assess the bidirectional relationship between variables. A directional assessment was made through mediation analysis, inputting the BCE score as an independent variable. Mediators included Faith in Humanity, Humanism, and Kantianism; Flourishing was set as the model’s final outcome.

To validate the regression models within the mediation framework, Goldfeld-Quandt and Harrison-McCabe tests were employed to assess homoscedasticity. Both tests yielded high *p*-values (0.891 and 0.893, respectively), indicating no significant evidence of heteroscedasticity or model misspecification. Consequently, the regression model is deemed adequately specified, adhering to constant variance assumptions and appropriate functional form. The Durbin-Watson test statistic (*DW* = 2.0739, *p* = 0.426) revealed no significant evidence of autocorrelation in the residuals; the assumption of independent errors in the regression model is supported. The Variance Inflation Factor (*VIF*) values for the predictors in the mediation model are as follows: BCE = 1.0922, Faith in Humanity = 1.4115, Humanism = 1.6656, and Kantianism = 1.4104. These values indicate low levels of multicollinearity among the predictors, as all VIF values are below the commonly accepted threshold of 10. Therefore, the mediation model is not significantly affected by multicollinearity. All hypotheses were tested at a 95% confidence level using Jamovi (The Jamovi Project, 2023).

## 3 Results

On average, respondents reported 7.34 (*SD* = 1.76) Benevolent Childhood Experiences. The number of reported BCEs did not vary significantly between men (*M* = 7.47; SD = 1.56) and women (*M* = 7.26; *SD* = 1.87); *t* = 121, *p* = 0.226, *d* = −0.12. However, specific BCEs were categorically associated with subjects’ sex. For instance, having at least one teacher who cared about the respondent (*χ^2^ =* 4.28*, p =* 0.039*, CC = 0*.10), having opportunities to have a good time (*χ^2^ =* 8.84*, p =* 0.003*, CC = 0*.15), and liking/feeling comfortable with oneself (*χ^2^ =* 5.88*, p =* 0.015*, CC = 0*.12); with a higher proportion of men reporting the presence of mentioned BCE, see Table 1.

**Table 1:** Prevalence of BCE compared between men and women.

Variable	Global		Female		Male		Contrast		
	Yes	No	Yes	No	Yes	No	*χ^2^*	*p*	*CC*
BCE 1. At least one caregiver with whom you felt safe	336 (82%)	74 (18%)	216 (85%)	39 (15%)	120 (77%)	35 (23%)	3.46	0.063	0.09
BCE 2. At least one good friend	352 (86%)	58 (14%)	216 (85%)	39 (15%)	136 (88%)	19 (12%)	0.73	0.392	0.04
BCE 3. Beliefs that comforted you	349 (85%)	61 (15%)	218 (85%)	37 (15%)	131 (85%)	24 (15%)	0.07	0.788	0.01
BCE 4. Liked going to school	375 (91%)	35 (9%)	231 (91%)	24 (9%)	144 (93%)	11 (7%)	0.66	0.416	0.04
BCE 5. At least one teacher who cared for you	296 (72%)	114 (28%)	175 (69%)	80 (31%)	121 (78%)	34 (22%)	4.28	**0.039**	0.1
BCE 6. Good neighbors	312 (76%)	98 (24%)	189 (74%)	66 (26%)	123 (79%)	32 (21%)	1.45	0.228	0.06
BCE 7. Had an adult who supported or advised you	304 (74%)	106 (26%)	194 (76%)	61 (24%)	110 (71%)	45 (29%)	1.31	0.252	0.06
BCE 8. Opportunities to have a good time	379 (92%)	31 (8%)	228 (89%)	27 (11%)	151 (97%)	4 (3%)	8.84	**0.003**	0.15
BCE 9. Liked yourself or felt comfortable with yourself	283 (69%)	127 (31%)	165 (65%)	90 (35%)	118 (76%)	37 (24%)	5.88	**0.015**	0.12
BCE 10. Predictable home routine	307 (75%)	103 (25%)	185 (73%)	70 (27%)	122 (79%)	33 (21%)	1.95	0.163	0.07

Significant *p*-values (<0.05) are presented in bold. CC, Contingency Coefficient.

Flourishing was significantly higher for participants who reported the presence of BCEs (*p* < 0.05); this holds for all individual BCE items included in the study, see Table 2. The largest effect size was achieved for the difference in Flourishing scores between those who reported they liked going to school as a child and those who disliked it (*d* = −1.17). Medium effect sizes were detected for the following indicators (*d* > |0.50|): having at least one good friend (*d* = −0.62), good neighbors (*d* = −0.63), having comfortable beliefs (*d* = −0.75), liking/feeling comfortable with oneself (*d* = −0.70), and having a predictable home routine (*d* = −0.58).

**Table 2:** Comparisons in Flourishing and Light Triad scores based on BCE experiences.

BCE	Variable	Response	*n*	Mean	*SD*	*t*	*p*	*d*
BCE 1. At least one caregiver with whom you felt safe	Flourishing	Yes	74	41.92	11.2	−2.3	**0.02**	−0.31
		No	336	45.18	9.58			
	Faith in Humanity	Yes	74	13.41	3.57	−0.7	0.5	−0.09
		No	336	13.71	3.42			
	Humanism	Yes	74	15.85	3.66	−2.4	**0.02**	−0.31
		No	336	16.97	3.45			
	Kantianism	Yes	74	14.89	3.41	−1.6	0.12	−0.2
		No	336	15.58	3.31			
BCE 2. At least one good friend	Flourishing	Yes	58	39.48	9.45	−4.4	**< 0.001**	−0.62
		No	352	45.43	9.8			
	Faith in Humanity	Yes	58	12.4	3.72	−2.8	**0.01**	−0.41
		No	352	13.86	3.36			
	Humanism	Yes	58	15.78	3.99	−2.1	**0.04**	−0.31
		No	352	16.93	3.4			
	Kantianism	Yes	58	15.31	3.28	−0.4	0.72	−0.05
		No	352	15.48	3.35			
BCE 3. Beliefs that comforted you	Flourishing	Yes	61	38.23	10.6	−5.2	**< 0.001**	−0.75
		No	349	45.7	9.43			
	Faith in Humanity	Yes	61	12.59	3.4	−2.7	**0.01**	−0.37
		No	349	13.84	3.42			
	Humanism	Yes	61	16.03	3.64	−1.7	0.09	−0.24
		No	349	16.89	3.48			
	Kantianism	Yes	61	14.92	3.02	−1.5	0.15	−0.2
		No	349	15.55	3.39			
BCE 4. Liked going to school	Flourishing	Yes	35	33.49	11.4	−6.1	**< 0.001**	−1.17
		No	375	45.63	9.16			
	Faith in Humanity	Yes	35	10.77	3.62	−5	**< 0.001**	−0.91
		No	375	13.93	3.31			
	Humanism	Yes	35	14.23	4.47	−3.6	**< 0.001**	−0.7
		No	375	17	3.32			
	Kantianism	Yes	35	13.77	4.6	−2.3	**0.03**	−0.47
		No	375	15.61	3.16			
BCE 5. At least one teacher who cared for you	Flourishing	Yes	114	41.41	11	−3.8	**< 0.001**	−0.43
		No	296	45.81	9.25			
	Faith in Humanity	Yes	114	13.34	3.53	−1.1	0.26	−0.13
		No	296	13.78	3.41			
	Humanism	Yes	114	16.48	3.57	1	0.32	−0.11
		No	296	16.88	3.49			
	Kantianism	Yes	114	15.38	3.45	−0.3	0.78	−0.03
		No	296	15.48	3.3			
BCE 6. Good neighbors	Flourishing	Yes	98	39.8	10.6	−5.3	**< 0.001**	−0.63
		No	312	46.1	9.27			
	Faith in Humanity	Yes	98	12.41	3.64	−4	**< 0.001**	−0.47
		No	312	14.05	3.29			
	Humanism	Yes	98	16.14	3.68	−2	0.05	−0.23
		No	312	16.96	3.44			
	Kantianism	Yes	98	15.15	3.39	−1	0.31	−0.12
		No	312	15.55	3.32			
BCE 7. Adult who supported or advised you	Flourishing	Yes	106	41.12	11.4	−3.8	**< 0.001**	−0.45
		No	304	45.8	9.11			
	Faith in Humanity	Yes	106	12.6	3.55	−3.6	**< 0.001**	−0.41
		No	304	14.02	3.34			
	Humanism	Yes	106	15.93	3.74	−2.7	**0.01**	−0.31
		No	304	17.06	3.38			
	Kantianism	Yes	106	14.86	3.5	−2.1	**0.04**	−0.24
		No	304	15.66	3.26			
BCE 8. Opportunities to have a good time	Flourishing	Yes	31	39.52	12.4	−2.4	**0.02**	−0.49
		No	379	45.01	9.63			
	Faith in Humanity	Yes	31	12.29	3.8	−2.1	**0.04**	−0.41
		No	379	13.77	3.4			
	Humanism	Yes	31	15.94	4.11	−1.2	0.25	−0.24
		No	379	16.83	3.45			
	Kantianism	Yes	31	14.65	4.22	−1.1	0.27	0.23
		No	379	15.52	3.25			
BCE 9. Liked yourself or felt comfortable with yourself	Flourishing	Yes	127	39.92	10.3	−6.4	**< 0.001**	−0.7
		No	283	46.69	9.06			
	Faith in Humanity	Yes	127	12.73	3.43	−3.7	**< 0.001**	−0.39
		No	283	14.07	3.38			
	Humanism	Yes	127	16.3	3.53	−1.8	0.07	−0.19
		No	283	16.98	3.49			
	Kantianism	Yes	127	15.39	3.24	−0.3	0.81	−0.03
		No	283	15.48	3.39			
BCE 10. Predictable home routine	Flourishing	Yes	103	40.26	10.7	−4.9	**< 0.001**	−0.58
		No	307	46.04	9.28			
	Faith in Humanity	Yes	103	12.47	3.55	−4.00	**< 0.001**	−0.46
		No	307	14.06	3.32			
	Humanism	Yes	103	16.13	4.05	−1.9	0.06	−0.23
		No	307	16.98	3.29			
	Kantianism	Yes	103	15.05	3.24	−1.5	0.15	−0.16
		No	307	15.59	3.37			

Significant *p*-values (<0.05) are presented in bold.

On the other hand, Faith in Humanity scores are higher for those who reported the presence of having at least one good friend (*d* = −0.41), comfortable beliefs (*d* = −0.37), liking school (*d* = −0.91), good neighbors (*d* = −0.47), having an adult supporter/adviser (*d* = −0.41), opportunities for a good time (*d* = −0.41), liking/feeling comfortable with oneself (*d* = −0.39), and having a predictable home routine (*d* = −0.46). Humanism and Kantianism were higher for those who liked attending school and had an adult advisor/supporter.

The number of reported BCEs correlates positively and significantly with Flourishing (*r* = 0.43; *p* < 0.001) and all the Light Triad traits (*p* < 0.01). Flourishing scores also have significant positive associations with the Light Triad (*p* < 0.001), see Table 3.

**Table 3:** Bidirectional correlations between variables.

Variable	Statistic	BCE	Flourishing	Faith in Humanity	Humanism	Kantianism
BCE	Pearson’s *r*	—				
	*p*-value	—				
	*CI UL* 95%	—				
	*IC LL* 95%	—				
Flourishing	Pearson’s *r*	0.43^***^	—	0.54^***^	0.51^***^	0.35^***^
	*p*-value	< 0.001	—	< 0.001	< 0.001	< 0.001
	*CI UL* 95%	0.50	—	0.61	0.58	0.43
	*IC LL* 95%	0.35	—	0.47	0.44	0.26
Faith in Humanity	Pearson’s *r*	0.28^***^	0.54^***^	—		
	*p*-value	< 0.001	< 0.001	—		
	*CI UL* 95%	0.37	0.61	—		
	*IC LL* 95%	0.19	0.47	—		
Humanism	Pearson’s *r*	0.21^***^	0.51^***^	0.50^***^	—	
	*p*-value	< 0.001	< 0.001	< 0.001	—	
	*CI UL* 95%	0.30	0.58	0.57	—	
	*IC LL* 95%	0.12	0.44	0.43	—	
Kantianism	Pearson’s *r*	0.13^**^	0.35^***^	0.35^***^	0.53^***^	—
	*p*-value	0.009	< 0.001	< 0.001	< 0.001	—
	*CI UL* 95%	0.22	0.43	0.43	0.60	—
	*IC LL* 95%	0.03	0.26	0.26	0.46	—

^**^ *p* < 0.01, ^***^ *p* < 0.001.

The number of BCE had a significant total (*β* = 0.43; *p* < 0.001) and direct effect (*β* = 0.28; *p* < 0.001) on Flourishing scores. However, significant indirect effects were also identified. Faith in Humanity (*β* = 0.09, *p* < 0.001) and Humanism (*β* = 0.06, *p* < 0.001), not Kantianism (*β* = 0.01, *p* = 0.197) mediated the relationship between BCE and Flourishing. At a component level, BCE score significantly explained all Light Triad traits (*p* < 0.01). Faith in Humanity (*β* = 0.31, *p* < 0.001) and Humanism (*β* = 0.27, *p* < 0.001), not Kantianism (*β* = 0.06, *p* = 0.139), directly explained Flourishing scores. Overall, the model accounted for 45% of the variance in Flourishing, see Table 4.

**Table 4:** Mediation analysis of BCE effects on Flourishing.

Type	Effect	Estimate 95% *CI* [*LL, UL*]	*SE*	*β*	*z*	*p*
Indirect	BCE ⇒ Faith in Humanity ⇒ Flourishing	0.48 [0.27, 0.69]	0.11	0.09	4.51	**< 0.001**
	BCE ⇒ Humanism ⇒ Flourishing	0.32 [0.14, 0.50]	0.09	0.06	3.44	**< 0.001**
	BCE ⇒ Kantianism ⇒ Flourishing	0.05 [−0.02, 0.12]	0.04	0.01	1.29	0.197
Component	BCE ⇒ Faith in Humanity	0.55 [0.36, 0.73]	0.09	0.28	5.88	**< 0.001**
	Faith in Humanity ⇒ Flourishing	0.89 [0.64, 1.13]	0.13	0.31	7.02	**< 0.001**
	BCE ⇒ Humanism	0.42 [0.23, 0.61]	0.10	0.21	4.36	**< 0.001**
	Humanism ⇒ Flourishing	0.76 [0.49, 1.02]	0.13	0.27	5.63	**< 0.001**
	BCE ⇒ Kantianism	0.24 [0.06, 0.43]	0.09	0.13	2.63	**0.009**
	Kantianism ⇒ Flourishing	0.19 [−0.06, 0.45]	0.13	0.06	1.48	0.139
Direct	BCE ⇒ Flourishing	1.57 [1.15, 2.00]	0.22	0.28	7.24	**< 0.001**
Total	BCE ⇒ Flourishing	2.42 [1.93,2.92]	0.25	0.43	9.59	**< 0.001**

Betas are standardized effect sizes. LL, Lower Limit; UL, Upper Limit. Significant *p*-values (<0.05) are presented in bold.

## 4 Discussion

The results of this study have several theoretical and practical implications for the field of positive psychology. First, the findings suggest that Benevolent Childhood Experiences are positively associated with Flourishing and the Light Triad traits. The Light Triad traits are considered positive personality characteristics that include Kantianism (respecting the dignity of all individuals), Humanism (valuing the well-being of others), and Faith in Humanity (believing in the inherent goodness of people). The fact that BCEs have a significant positive relationship with these traits underscores the importance of a positive childhood environment in fostering healthy personality traits.

Moreover, the study reveals that BCEs directly affect Flourishing scores. BCEs, such as having good friends or liking school, were associated with higher Flourishing, indicating that a positive childhood environment is important for one’s overall well-being. Furthermore, the indirect effects of BCEs on Flourishing via Faith in Humanity and Humanism suggest that these personality traits may mediate between BCEs and Flourishing.

Individuals reporting BCEs may develop greater Faith in Humanity and hold positive perceptions of others due to several potential underlying mechanisms. First, BCEs involving nurturing relationships and support from caregivers may foster a sense of trust and security, leading individuals to view others as reliable and compassionate. Second, positive childhood experiences can contribute to developing a positive self-concept, allowing individuals to project their positive attributes onto others and perceive them favorably. Third, BCEs may enhance individuals’ social and emotional competence, promoting empathy and understanding toward others, and reinforcing the belief in the inherent goodness and value of people (AlShawi and Lafta, 2015; Lekaviciene and Antiniene, 2016; Berduzco-Torres et al., 2020; Streit et al., 2020). These factors collectively contribute to a more positive worldview.

The examination of Kantianism within the Light Triad framework in this study has provided valuable insights, although conclusive results remain elusive. While a positive correlation between Kantianism and Flourishing implies a possible connection between the concept of inherent human worth and well-being, the lack of a significant direct effect and its comparatively weaker mediating role, in contrast to Faith in Humanity and Humanism, calls for deeper scrutiny. The varying effects of Kantianism, Faith in Humanity, and Humanism on Flourishing are likely influenced by their unique cognitive intricacies and socialization mechanisms. Faith in Humanity and Humanism are likely shaped by positive interpersonal experiences and cultural norms promoting empathy (Neumann et al., 2020; Ramos-Vera et al., 2023), while Kantianism requires individuals to engage in complex moral reasoning, which may not directly correlate with self-reported well-being. Furthermore, differences in measurement accuracy could contribute to these observed variations, with Kantianism posing particular challenges for assessment. By gaining a deeper understanding of the mechanisms underlying Kantianism’s influence on Flourishing, we can refine interventions aimed at enhancing well-being.

On the other hand, the fact that specific BCEs were associated with subjects’ sex indicates that there may be sex-based differences in how individuals experience and perceive their childhood environment. For example, a higher proportion of men reported having opportunities to have a good time or liking/feeling comfortable with themselves as BCEs. Additionally, previous studies suggest that Honduran women have experienced more ACEs than men (Kappel et al., 2021). Therefore, public interventions should aim to minimize the difference between boys and girls in terms of BCEs.

The study also has practical implications for clinical psychology and schools. The results suggest that interventions promoting BCEs, such as fostering positive relationships with teachers, creating opportunities for socializing, or promoting a sense of comfort and acceptance, may contribute to positive mental health and well-being outcomes. The findings suggest that schools may play a crucial role in promoting a positive childhood environment by providing opportunities for socialization and positive relationships with teachers.

School support provides a nurturing environment that enhances a child’s emotional intelligence, sense of belonging, competence, and positive relationships, foundational elements for healthy psychological development (Puertas Molero et al., 2020; Gramaxo et al., 2023). Liking school reflects positive experiences, engagement, and a sense of purpose, contributing to intrinsic motivation and developing a positive worldview. These positive school experiences can foster social–emotional competencies (Graham et al., 2022), including empathy and prosocial behaviors, which in turn cultivate Faith in Humanity and the perception of people as inherently valuable and deserving of dignity. Such early experiences lay the groundwork for positive adult outcomes, contributing to overall Flourishing and forming positive beliefs about Humanity. This highlights the importance of incorporating socioemotional education within the school setting (Govorova et al., 2020) and universal positive mental health screenings (Cortés-Ramos and Landa-Blanco, 2021).

In this sense, previous research suggests that school connectedness is linked to reducing students’ anxiety and depression symptoms (Raniti et al., 2022). Since teachers play an important role in forming BCEs, public policy should also target teachers’ well-being to promote students’ mental health. In this sense, teacher well-being has been associated with higher student well-being and lower distress scores (Harding et al., 2019).

Several limitations should be considered when interpreting the findings of this study. First, the use of self-reported data is susceptible to recall bias and social desirability bias. Participants may have had difficulty accurately recalling their childhood experiences, potentially leading to inaccuracies in reporting BCEs, Light Triad traits, and Flourishing. Second, the study’s reliance on a non-probabilistic sampling method limits the generalizability of the findings to the broader population of Honduran adults. The sample may not represent the entire population, and the results may not apply to individuals with different socio-demographic characteristics or from other cultural backgrounds. Replication of our findings across diverse populations is crucial for assessing robustness. Third, while we employed a mediation model for analysis, which yielded informative results, its ability to fully capture the complexity of these relationships may be limited. In future research, utilizing Structural Equation Models (SEM) could offer a more comprehensive understanding by better controlling for predictors and mediators. Fourth, although our study identified significant direct and indirect effects of BCEs on Flourishing, the model’s explanatory power was modest. Future research could explore additional factors to elucidate the pathways through which BCEs influence Flourishing. Fifth, our study’s exclusive focus on quantitative data overlooks qualitative perspectives that could provide deeper insights into participants’ experiences. Incorporating qualitative research methods could enrich our understanding of the underlying mechanisms.

In light of these limitations, future research endeavors should leverage more sophisticated statistical techniques, integrate qualitative methodologies, and expand the scope of investigation to deepen our understanding of these complex constructs. Additionally, longitudinal studies are essential for uncovering temporal relationships and comprehending the developmental trajectories of BCEs, Light Triad traits, and Flourishing. Exploring the interaction of Kantianism with other personality traits and cultural contexts could provide valuable insights into promoting positive outcomes in adulthood and enhancing Flourishing.

In conclusion, this study underscores the significance of Benevolent Childhood Experiences in fostering Flourishing and positive personality traits, particularly highlighting the potential of the Light Triad framework in understanding well-being. While the specific role of Kantianism warrants further investigation, Faith in Humanity and Humanism emerge as pivotal pathways for nurturing Flourishing through positive childhood experiences. Moving forward, it is crucial for future research to assess the long-term societal benefits of policies aimed at promoting positive childhood experiences. Initiatives focusing on positive mental health, enhancing educational accessibility, and fostering intergenerational relationships hold promise for cultivating a more conducive childhood environment.

## Data availability statement

The raw data supporting the conclusions of this article will be made available by the authors, without undue reservation.

## Ethics statement

The studies involving humans were approved by Comité de Ética en Investigación de la Facultad de Ciencias Sociales (UNAH). The studies were conducted in accordance with the local legislation and institutional requirements. The participants provided their written informed consent to participate in this study.

## Author contributions

ML-B: Conceptualization, Formal analysis, Investigation, Methodology, Project administration, Supervision, Validation, Writing – original draft, Writing – review & editing. TH: Formal analysis, Investigation, Writing – original draft. HE: Formal analysis, Investigation, Writing – original draft. KG: Formal analysis, Investigation, Writing – original draft. SM: Formal analysis, Investigation, Writing – original draft. AC-R: Conceptualization, Methodology, Supervision, Validation, Writing – original draft.
